# Childhood socioemotional development and growth mindset preceding schizophrenia: case–control study using prospectively collected data

**DOI:** 10.1192/bjo.2026.11004

**Published:** 2026-04-06

**Authors:** Javiera Vasquez-Nuñez, José Conejeros, Camila Díaz-Dellarrossa, Cristian Mena, Juan Undurraga, Alfonso Gonzalez-Valderrama, Ruben Nachar, Susana Claro, Eduardo A. Undurraga, Nicolas A. Crossley

**Affiliations:** School of Government, Pontificia Universidad Católica de Chile, Santiago, Chile; Department of Psychology and Health Sciences, Universitat Autònoma de Barcelona, Barcelona, Spain; Department of Psychiatry, Pontificia Universidad Católica de Chile, Santiago, Chile; Surveillance, Epidemiology, and New Technologies for Infectious Emerging Threats (SENTINET), Santiago, Chile; Dr José Horwitz Barak Psychiatric Institute, Santiago, Chile; School of Medicine, Finis Terrae University, Santiago, Chile; Department of Neurology and Psychiatry, Universidad del Desarrollo-Clínica Alemana, Santiago, Chile; Millennium Nucleus for the Study of Early Mathematical Skill Development, Santiago, Chile; Centro de Interés Nacional para Investigación e Innovación en Niñez, Adolescencia, Resiliencia y Adversidad (IINARA), Santiago, Chile; Department of Psychiatry, University of Oxford, UK; Department of Psychiatry, University of Antioquia, Medellín, Colombia

**Keywords:** Premorbid, schizophrenia, self-esteem and motivation, parental support, growth mindset

## Abstract

**Background:**

Understanding premorbid socioemotional trajectories in schizophrenia is crucial for early identification and potential primary prevention. Studies in adults with schizophrenia suggest similar socioemotional premorbid difficulties, but are limited by their retrospective design.

**Aims:**

To contribute new insights into the premorbid socioemotional characteristics of schizophrenia, beyond the biases of retrospective studies, this research investigates three educational socioemotional dimensions in children who later developed the disorder.

**Method:**

We conducted a case–control study using prospectively collected data, examining childhood differences in perceived parental support, self-esteem and school motivation, and growth mindset (intelligence can improve through effort) among individuals who later developed schizophrenia (*n* = 341) and their classmates (*n* = 20 567). We constructed *z*-normalised indicators based on standardised national tests administered in fourth, eighth and tenth grades in Chile. Mixed linear models accounted for repeated measures and adjusted for educational level, gender, grade point average, school and year.

**Results:**

Children later diagnosed with schizophrenia reported less parental educational support compared with their classmates (*β* = −0.276, 95% CI −0.388 to −0.163). Only girls who later developed schizophrenia reported lower self-esteem and school motivation than their peers (*β* = −0.290, 95% CI −0.498 to −0.131). Contrary to our hypothesis, children who later developed schizophrenia showed a higher growth mindset (*β* = 0.287, 95% CI 0.077–0.497).

**Conclusions:**

Our results suggest that premorbid socioemotional characteristics in schizophrenia are detectable in childhood and may vary by gender. These findings highlight the potential of educational settings as platforms for preventive interventions aimed at enhancing parental support and monitoring students’ psycho-emotional well-being, while acknowledging gender-specific developmental trajectories and heterogeneity in premorbid functioning.

Schizophrenia imposes a substantial burden of disease and economic costs on individuals, communities and healthcare systems, ranking among the top 20 leading causes of global disability.^1^ Despite substantial advances in diagnosis and treatment, there remains a need for more effective therapies to support recovery (tertiary prevention), facilitate early identification (secondary prevention) and, ideally, prevent its onset (primary prevention).

One of the most widely accepted theories conceptualises schizophrenia as a neurodevelopmental disorder, with early developmental alterations preceding the manifestation of psychotic symptoms in late adolescence,^2^ underscoring the importance of investigating the period preceding the manifestation of psychotic symptoms. Several studies have focused on cognitive function or indicators of educational performance before the onset of the disorder.^3^ Other aspects of the premorbid stage have also been studied, such as socioemotional functioning, which often relies on retrospective accounts that have recognised limitations.^4^ Prospective studies looking at birth cohorts and other sources of data have shown that young people who later develop schizophrenia score high in neuroticism,^5^ have difficulties maintaining close personal relationships^6^ and display social withdrawal.^6,7^ Further understanding of the social and emotional precursors of psychosis could illuminate areas needing additional improvement or reinforcement.^8,9^

Most research on premorbid functioning in schizophrenia relies on retrospective reports, which are prone to memory bias and post-diagnostic reinterpretation. Our study uses prospectively collected, large-scale, school-age data to examine socioemotional traits before symptom onset. By assessing self-esteem, school motivation and growth mindset, constructs rarely studied prospectively in relation to later psychosis, we provide new insights into developmental precursors of severe mental illness. The importance of studying family functioning in the premorbid phase is supported by evidence suggesting that people diagnosed with schizophrenia report family difficulties before they become unwell with psychosis.^6,7,10^ Family-focused support has proven effective for relapse prevention and improving functional outcomes in schizophrenia,^7^ and could potentially prevent the development of psychosis.^7^ Low self-esteem is common among those at high clinical risk of psychosis,^11^ and is linked to delusion development and maintenance.^12^ Additionally, low perceived self-efficacy has been associated with decreased performance in social and daily functioning, and increased defeatist beliefs among persons with schizophrenia.^13^ Promoting self-efficacy and self-esteem in children could potentially enhance well-being and reduce the future risk of psychosis and other mental health problems.^11^ Finally, a growth mindset is defined as the belief that intelligence and learning ability can change over time through effort, perseverance and practice.^14^ A high growth mindset is related to higher learning, both measured by grade point average (year GPA) and standardised test scores.^15^ Importantly, it has also been associated with common mental health disorders in young people.^16^ Growth mindset has been associated with lower levels of depression and anxiety in young people. Unlike a fixed mindset, which is linked to feelings of helplessness and poor coping, a growth mindset promotes more adaptive interpretations of stressors such as social rejection, family conflict and academic failure.^16^ Growth mindset may be relevant to understanding the poorer academic performance consistently observed in individuals who later develop schizophrenia spectrum disorders.^17^ It has also been linked to greater self-efficacy, which may reduce vulnerability to internalising and externalising symptoms.^18^ These associations position growth mindset as a promising target for early mental health interventions, which has led to the exploratory integration of growth mindset interventions into support packages for young people at risk of psychosis.^19^

To shed further light on the premorbid period of schizophrenia, specifically the premorbid socioemotional characteristics, we examined three socioemotional dimensions in a cohort of 341 children who later developed schizophrenia, alongside 20 567 school classmates as controls: (a) self-esteem and school motivation, (b) perceived educational parental support and (c) growth mindset. The children were evaluated at ages 9, 13 and 15 years. These measures were obtained from government-sponsored annual nationwide educational and socioemotional progress tests. They were selected based on prior evidence supporting their role and because they could be a potential target for action. We hypothesised that children subsequently diagnosed with schizophrenia would demonstrate lower levels of self-esteem and school motivation, perceived educational parental support and a fixed mindset compared with their peers.

## Method

### Study population and design

We conducted a case–control study looking at differences ascertained during childhood in perceived parental support, self-esteem and school motivation and growth mindset in people who later developed schizophrenia compared with their classmates.

The group of children who later developed schizophrenia was identified from two registers where they were included by their treating psychiatrist after being clinically diagnosed with schizophrenia between 2007 and 2020, in Chile: (a) they were notified with a first diagnosis of schizophrenia according to Chilean law^20^ at the Instituto Psiquiátrico Dr. José Horwitz Barak, a tertiary psychiatric public hospital in Northern Santiago; or (b) they were registered with the National Clozapine Hematological Monitoring System at the Instituto Psiquiátrico Horwitz when they were started on clozapine by their treating team anywhere in Chile.^21^ All participants in these registers for whom data were available for at least one question on educational indicators of personal and social development in at least 1 year between 2012 and 2019 were included. Children may have no data on these questions because of non-attendance at school on the day of the test or non-response to these questions.

The control group consisted of classmates of the participants identified as later developing schizophrenia who attended the same school and year at the time of the assessment.

We used anonymised data from the System for the Measurement of the Quality of Education (SIMCE) on students’ characteristics and test scores. The SIMCE is a standardised government test applied annually to all students in fourth grade (9 years old) and on alternate years to those in eighth (13 years old) and tenth grade (15 years old). Data were obtained from tests administered between 2012 and 2019. Some participants may have taken the test more than once. For example, in the test administered in 2012, they were 9 years old, and in the test administered in 2018, they were 15 years old. Assessments include parental and household data from the SIMCE Complementary Survey for Parents, which collects information on students’ social and personal development, as well as administrative data from the Ministry of Education and the National Institute of Statistics.

To merge the education and health databases, we implemented a system that prevented the disclosure of health-related information to those managing the identifiable educational database. At the same time, health researchers were provided with pseudoanonymised data, preventing them from linking educational results with a specific individual. Details of the procedure are in the Supplementary Fig. 1 available at https://doi.org/10.1192/bjo.2026.11004. As approved by the ethics committee, consent was not obtained from participants as analyses were based on anonymised data.

### Ethics

The study procedures complied with national ethical guidelines and the 2013 revision of the Helsinki Declaration. All procedures complied with Chilean ethical and legal regulations and were approved by the Institutional Ethical Scientific Committee (approval number AE-010/2021). Researchers accessed only anonymised data provided through secure governmental channels. The anonymisation process, which ensured that no identifiable information was accessible to the research team or members of the Ministry of Education, and that the clinical team received only group-level information about scholastic achievement of the patients, is detailed in Supplementary Fig. 1. As authorised by the ethics committee, consent was not obtained from participants as analyses were based on anonymised data; the waiver of consent was justified based on minimal risk, impracticability and public interest.

### Outcomes and covariates

We constructed three indicators based on the questionnaires applied focusing on (a) self-esteem and school motivation, (b) perceived educational parental support and (c) growth mindset. Our measures of academic self-esteem, school motivation and growth mindset were developed or adapted by the National Agency for the Quality in Education as mandated by the Chilean Ministry of Education as part of the national SIMCE assessments required by the Law of the Quality Assurance System (SAC in Spanish). Instrument development followed rigorous procedures to ensure validity, reliability and cultural adaptation, including literature reviews, qualitative fieldwork, expert consultations and pilot testing in representative samples. Items were evaluated through cognitive interviews and statistical analyses, and the instruments were informed by internationally validated scales (e.g. Rosenberg Self-Esteem Scale, Self-Description Questionnaire, Student Motivation and Engagement Scale) adapted for the Chilean context.^22^

The Education Quality Agency did not use the same set of questions for these indicators each year, so we calculated a standardised *z*-score for each student and variable by using the indicators available each year and comparing them to their classmates. We performed an exploratory factor analysis, selecting questions that loaded on the same dimension to maximise the internal consistency of the construct, using Cronbach’s *α* (details of internal consistency in Supplementary Tables 1–3). This approach allowed for a direct comparison of cases with their classmates across years and different schools, using all the available data for each outcome (details on the questions used each year are in Supplementary Tables 1(a), 2(a) and 3(a). All available questions were answered on a four-category Likert scale (**‘**strongly agree’, **‘**agree’, **‘**disagree’, ‘strongly disagree’).

We standardised psychosocial scores within schools, allowing for interpretation relative to each school cohort. Since the Chilean education system is highly segregated across economic groups,^23^ this also reduces between-school variance linked to structural socioeconomic status differences. Multilevel models also included random intercepts for schools, further adjusting for unobserved contextual factors such as socioeconomic differences between schools. Although direct individual socioeconomic status indicators to control for intra-school variance were unavailable because of missing data, this approach offers a partial control for socioeconomic variation.

Self-esteem and school motivation were built from questions that addressed students’ self-perception and self-assessment of their learning ability, possibilities for self-improvement, assessment of their academic attributes and skills, willingness to learn, attitudes toward study difficulties and achievement motivation. Although the concepts of self-esteem and school motivation measure different psychological characteristics, these concepts are strongly correlated.^24^ Evidence suggests that students with higher self-esteem show greater resilience and perseverance in the face of challenges, enabling them to be more motivated in their studies.^25^ This has also been considered by the Chilean Education Quality Agency, which treats them as a single indicator of socioemotional development. Our factor analyses confirmed that a one-factor structure provided the best fit and highest reliability (Cronbach’s *α* = 0.74–0.89). Therefore, this composite reflects both theoretical and empirical considerations. An example of these is, **‘**I know I can always do my homework well’.

Perceived educational parental support was built from questions about students’ perceived support from parents, such as **‘**My parents help me study’. As reported previously,^26^ growth mindset was measured using the level of agreement or disagreement with statements that together reflect children’s disposition toward the belief that intelligence is either a fixed and unchangeable trait or a malleable process that can be improved through hard work, practice and experience. An example of this indicator is **‘**Intelligence is something that cannot be changed much’. The three indicators were coded so that lower scores indicated lower self-esteem and school motivation, lower perceived educational parental support and a fixed mindset.

An attrition analysis compared participants with and without socioemotional data by gender and GPA. Details of this analysis are in Supplementary Fig. 2. Missing data were mainly attributable to the structure of the national assessments, which varied by school level and year. Growth mindset was assessed only in selected survey waves, yielding a smaller analytic sample. GPA was adjusted for in all mixed-effects models.

We included several variables that could confound a possible association, including gender, school level, clozapine prescription and year GPA. The latter was a *z*-score indicating the student’s performance according to the average grade in their school in the same year.

### Statistical analysis

We constructed three independent linear mixed models to determine the association between the future development of schizophrenia with (a) self-esteem and school motivation, (b) perceived educational parental support and (c) growth mindset. We included gender, level and academic performance in our models. To account for the nested structure of the data, unobserved characteristics of the school attended were controlled for by using random intercepts at the school level. The same students were assessed at multiple time points (fourth, eighth and tenth grades), but not all contributed data at every wave, because of school mobility and incomplete participation. To account for this unbalanced longitudinal structure and the correlation of repeated measures within individuals, we used mixed-effects models including random effects at the individual level. The model thus had the form of:

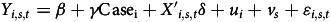




where *i* indexes individuals, *s* indexes schools and *t* indicates time. Case is a binary independent variable of interest (Case = 1, schizophrenia) with *γ* as the coefficient, *X*′_*i*, *s*, *t*_ is a vector of covariates used as controls (gender, level, year GPA, school), *δ* is the vector of coefficients accompanying the covariates at the level of the individual, *u*_*i*_ is the individual-level random effect (variability across repeated measures for each individual), *v*_*s*_ represents the school-level random intercepts (school effects shared by individuals within the school) and *ϵ*_*i*, *s*, *t*_ is the residual error term.

Two further analyses examined specific aspects. First, to examine whether the association between schizophrenia and our primary outcomes varied by the gender of the child, we added interaction terms (Case_*i*_ × Gender_*i*_). Second, we explored whether any differences found were driven by the subgroup of patients receiving clozapine, which represented the most severely unwell patients, including an extra fixed-effect term in the above model (clozapine prescription). Sensitivity analyses are presented in Supplementary Table 5.

All statistical analyses were conducted with Stata (version 16 for Windows; StataCorp LLC, College Station, Texas, USA: www.stata.com); multilevel mixed-effects linear regressions were estimated with the *xtmixed* function. The graphs were generated with RStudio (2025.05 for Windows; Posit Software PBC, Boston, Massachusetts, USA; posit.co/download/rstudio-desktop).

## Results

### Characteristics sample

We included 341 children (117 girls) who subsequently developed schizophrenia, of whom 82.4% required clozapine treatment. Of them, 76 provided data in fourth grade, 148 in eighth grade and 214 in tenth grade. These children were compared to 20 567 (9813 girls) participants without a subsequently known diagnosis of schizophrenia who attended the same school and level that year (the mean number of health classmates per subject on each level and course was 32.34, and the median was 34). For all three outcomes, the sample used was unbalanced with respect to the gender of the students, with an over-representation of boys in the schizophrenia groups. Participants with and without socioemotional data showed minimal gender differences across most outcomes, whereas higher GPA was associated with data availability. For growth mindset, data availability differed by gender, reflecting its assessment in selected cohorts and school levels. Table 1 describes the sample used, and Supplementary Table 4 shows details on included and not included observations.

**Table 1:** Characteristics of study participants by educational level, Chile, 2012–2019

Characteristic	Total	Control	Schizophrenia
	*n* (% female)	*n* (% female)	*n* (% female)
All levels			
Total	20 908 (47.49)	20 567 (47.71)	341 (34.31)
Perceived educational parental support	14 187 (45.79)	13 917 (47.4)	270 (33.7)
Self-esteem and school motivation	20 893 (47.49)	20 552 (47.71)	341 (34.31)
Growth mindset	5555 (50.03)	5472 (50.33)	83 (30.12)
Clozapine prescription	---	0	281 (35.51)
Year school GPA	20 755 (47.51)	20 414 (47.73)	341 (34.31)
Sample level fourth grade (age: 9 years)			
Total	2955 (49.72)	2880 (49.93)	76 (42.1)
Perceived educational parental support	2590 (49.03)	2524 (49.17)	66 (43.94)
Self-esteem and school motivation	2955 (49.71)	2879 (49.91)	76 (42.11)
Growth mindset	0	0	0
Clozapine prescription	---	0	70 (42.86)
Year school GPA	2926 (49.79)	2850 (50)	76 (42.1)
Sample level eighth grade (age: 13 years)			
Total	6980 (48.24)	6832 (48.48)	148 (37.16)
Perceived educational parental support	4008 (47.33)	3921 (47.54)	87 (37.93)
Self-esteem and school motivation	6974 (48.25)	6826 (48.49)	148 (37.16)
Growth mindset	751 (51.66)	739 (52.1)	12 (25)
Clozapine prescription	---	0	119 (39.5)
Year school GPA	6928 (48.24)	6785 (48.45)	143 (38.46)
Sample level tenth (age: 15 years)			
Total	12 138 (46.72)	11 924 (47.03)	214 (29.44)
Perceived educational parental support	7942 (45.76)	7791 (46.09)	151 (28.48)
Self-esteem and school motivation	12 128 (46.73)	11 914 (47.04)	214 (29.44)
Growth mindset	4864 (49.88)	4788 (50.21)	76 (28.95)
Clozapine prescription	---	0	171 (31)
Year school GPA	11 968 (46.7)	11 758 (47)	210 (30)

GPA, grade point average.

### Perceived educational parental support

**Fig. 1:**
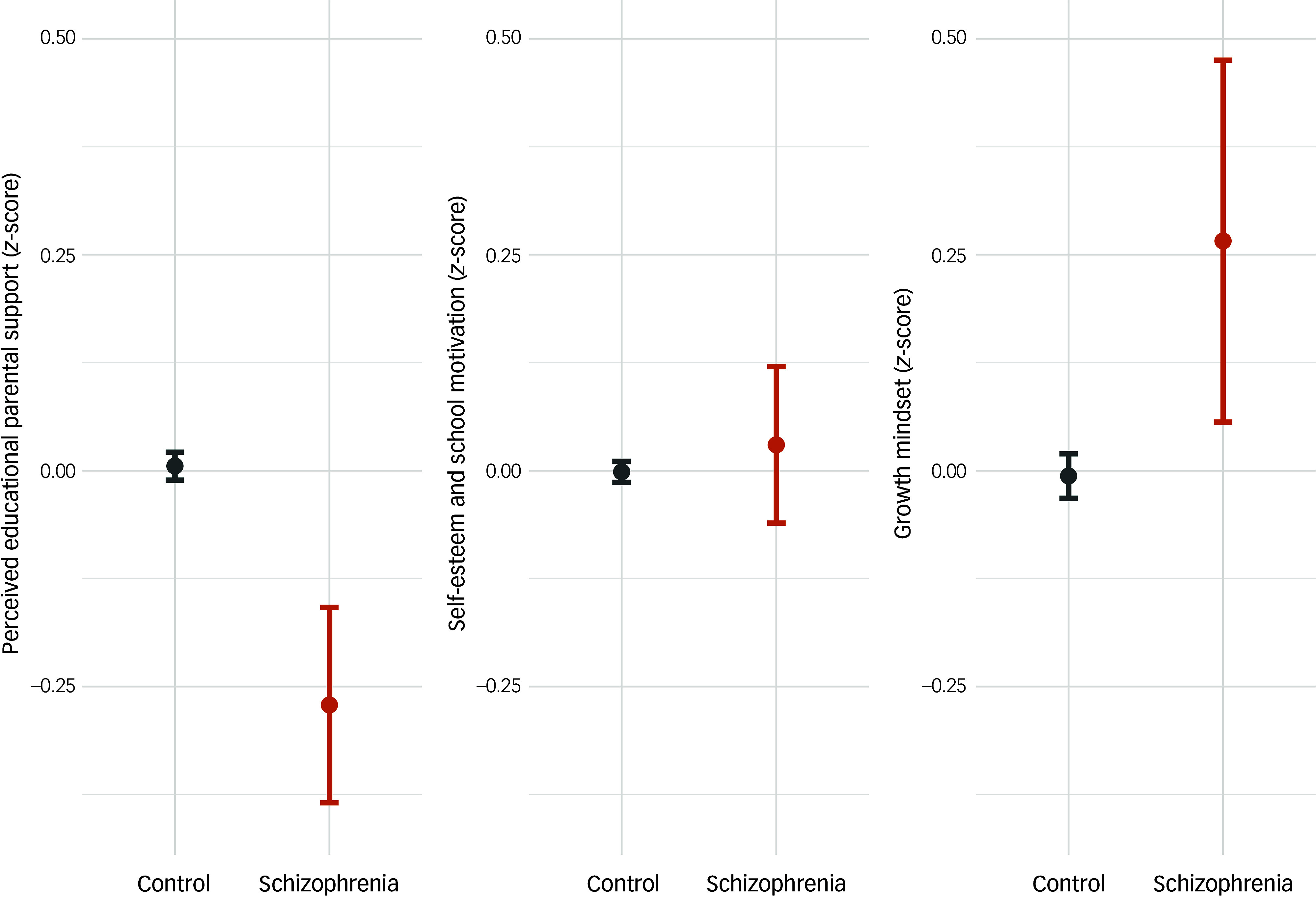
Perceived parental support, self-esteem and school motivation, and growth mindset in the premorbid phase. Standardised *z*-scores for each dimension among students later diagnosed with schizophrenia and those that have not received such diagnosis. Marginal effects are adjusted by mixed linear models, considering gender, students’ grade level, yearly grade point average, school and year. Error bars indicate 95% confidence intervals.

Perceived educational parental support was significantly lower in children who later developed schizophrenia (95% CI −0.404 to −0.178, *P* < 0.001; model 1A in Table 2). These children scored, on average, 0.3 s.d. lower than their healthy classmates, even after controlling for their GPA for the year (model 2A in Table 2 and Fig. 1). There was no significant interaction effect of gender in this outcome (model 1SA in Supplementary Table 5). We did not see a significant difference among children who later developed schizophrenia between those who were and were not prescribed clozapine (model 2SA in Supplementary Table 5).

**Table 2:** Mixed models for perceived educative parental support, self-esteem and school motivation, and growth mindset (cohort 2012–2019, fourth, eighth and tenth grades)

Variable	A. Perceived educational parental support		B. Self-esteem and school motivation		C. Growth mindset	
	Model 1A	Model 2A	Model 1B	Model 2B	Model 1C	Model 2C
Schizophrenia	−0.291**	−0.276**	−0.061	0.0441	0.262**	0.287**
	[−0.404 to −0.178]	[−0.388 to −0.163]	[−0.157 to 0.034]	[−0.044 to 0.132]	[0.050−0.473]	[0.077−0.49]
	(0.057)	(0.0575)	(0.0489)	(0.0450)	(0.108)	(0.107)
Female		−0.0242		0.0196		−0.066*
		[−0.056 to 0.008]		[−0.004 to 0.044]		[−0.117 to −0.014]
		(0.0165)		(0.0125)		(0.0263)
Fourth grade		0.0037		0.0010		
		[−0.041 to 0.048]		[−0.036 to 0.038]		
		(0.0231)		(0.0193)		
Eighth grade		0.0035		0.0054		0.0010
		[−0.038 to 0.045]		[−0.023 to 0.034]		[−0.089 to 0.091]
		(0.0213)		(0.0148)		(0.0462)
Year GPA		0.076**		0.403**		0.132**
		[0.059−0.093]		[0.390−0.415]		[0.106−0.158]
		(0.0086)		(0.0064)		(0.0134)
Constant	0.006	0.0121	0.0013	−0.0348*	−0.005	0.0156
	[−0.025 to 0.037]	[−0.026 to 0.050]	[−0.3 to 0.032]	[−0.068 to −0.00]	[−0.045 to 0.034]	[−0.031 to 0.062]
	(0.016)	(0.0197)	(0.016)	(0.0174)	(.020)	(0.0239)
Log-likelihood	−20 350.87	−20 311.3	−30 790.36	−28 986.74	−7793.71	−7745.46
AIC	40 719.75	40 650.59	61 604.73	58 007.47	15 603.42	15 514.92
Observations	14 540	14 540	22 057	22 057	5 615	5 615
Schools, *n*	417	417	550	550	165	165
Students, *n*	14 242	14 242	21 050	21 050	5 556	5 556
Variable: school (random intercept)	1.81e-07	2.13e-07	1.53e-11	1.30e-07	2.12e-08	1.56e-10
Variable: student (random intercept)	0.5330	0.5291	0.6756	0.5816	0.9636	0.9471
Residuals	0.8244	0.8238	0.7139	0.6925	0.1931	0.2222

GPA, grade point average; AIC, Akaike information criterion.

*n* = 14 540 for perceived educative parental support models, 22 057 for self-esteem and school motivation models, 5615 for growth mindset models. Robust standard errors are shown in parentheses. Coefficients reported correspond to unstandardised regression coefficients. 95% confidence intervals are shown in brackets.

*P < 0.05; **P < 0.001.

### Self-esteem and school motivation

**Fig. 2:**
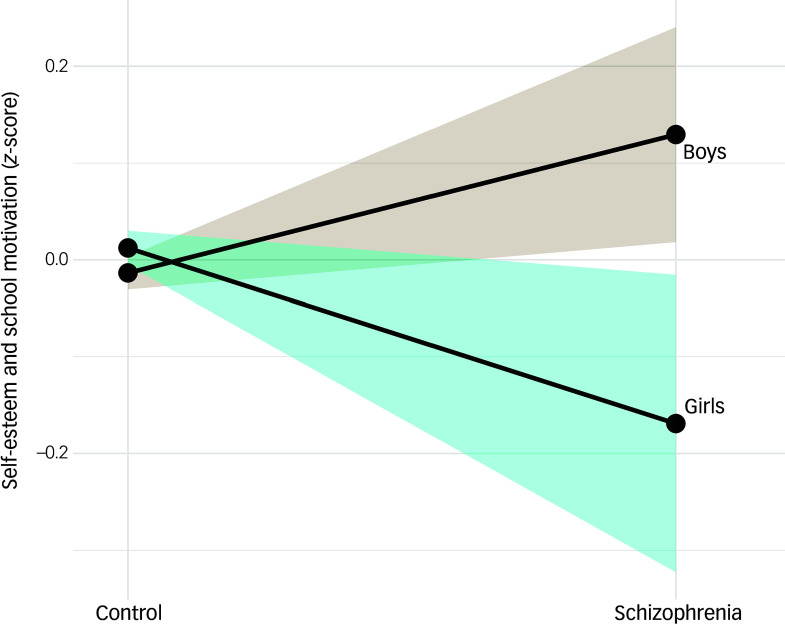
Interaction plot for self-esteem and school motivation showing the effect of diagnosis of schizophrenia and gender. The solid line and light turquoise confidence interval represent girls. The solid line and light brown confidence interval represent boys. The colour areas (light turquoise for girls and light brown for boys) represent the 95% confidence intervals.

We did not see a significant association between self-esteem and school motivation and future psychosis in our main model (Fig. 1). However, this relationship was significantly modulated by a case × gender interaction (95% CI −0.498 to −0.131, *P* < 0.001; model 1SB in Supplementary Table 5). In secondary analyses, we found that girls who later developed schizophrenia scored lower in self-esteem and school motivation compared with girls who did not (*β* = −0.290, *P* < 0.001), whereas the opposite was seen in boys (*β* = 0.153, *P* = 0.006; see Fig. 2 for interaction plot). We did not find any significant differences between those who developed treatment-resistant schizophrenia requiring clozapine and those who did not (model 2SB in Supplementary Table 5).

### Growth mindset

Finally, we found that children who later developed schizophrenia more frequently had a higher score on the growth mindset scale, suggesting a growth mindset instead of a fixed mindset compared with their peers across all model specifications. Specifically, these children scored on average 0.290 s.d. above their classmates on the growth mindset scale (95% CI 0.077−0.497, *P* = 0.007; Fig. 1 and model 2C in Table 2). In line with published literature, we also found that a growth mindset was associated with a higher year GPA (95% CI 0.106−0.158, *P* < 0.001; model 2C in Table 2), and girls had a slightly lower growth mindset than boys (95% CI −0.117 to −0.014, *P* = 0.012; model 2C in Table 2). Furthermore, we found a significant interaction between GPA and later schizophrenia diagnosis in predicting growth mindset scores (95% CI −0.356 to −0.046, *P* = 0.010; Supplementary Fig. 3). As shown in the figure, although higher academic achievement (GPA) was positively associated with growth mindset among youth in the control group, this relationship was reversed among children who later developed schizophrenia. In this group, higher GPA was associated with lower levels of growth mindset. Finally, the effect of psychosis on mindset was not significantly moderated by gender (model 1SC in Supplementary Table 5). Also, we did not find differences between children who were later treated with clozapine and those who were not (model 2SC in Supplementary Table 5).

## Discussion

Using a large sample of participants in Chile, this study provides estimates of the association between childhood premorbid educational self-esteem and motivation, perception of parental support and growth mindset in the period before developing schizophrenia. This study adds to the limited prospective research indicating that early socioemotional traits may signal vulnerability to psychosis. Prospective designs offer a less biased view of premorbid functioning than retrospective approaches.^4^ Our findings align with evidence that interpersonal and motivational deficits can precede schizophrenia by years, and highlight the potential of school-based psychosocial monitoring to identify risk during adolescence. We found that children who later developed schizophrenia reported lower perceived educational parental support and had a higher growth mindset than their peers. We also found that girls who later developed schizophrenia reported lower self-esteem and school motivation, whereas the opposite was seen in boys.

Our findings on perceived educational parental support align with a previous study examining family functioning among men with behavioural disorders who were undergoing the draft board assessment.^10,27,28^ Our results suggest that lower perceived educational parental support is not just present in those with premorbid behavioural disorders. This could be signalling a family dysfunction that could be either a contributing cause, a consequence of some of the premorbid alterations in the individual that disrupt the family dynamics,^10,27,28^ or a subjective negative perception of the individual, potentially influenced by subclinical or prodromal symptoms emerging close to adolescence. Retrospective studies have focused on reports of abnormal bonding with parental figures in the premorbid period. Regardless of the specific mechanism, our results highlight the importance of addressing the family environment in young people and the need to promote early interventions that strengthen positive parental and family bonding. Interventions that promote effective communication, conflict resolution and educational support have been shown to reduce stress in family dynamics among children, thereby reducing the likelihood of severe psychiatric disorders in the future.^27^

Low self-esteem and school motivation are central constructs in psychological theories explaining the path to schizophrenia from the premorbid period.^13^ Our results partially confirmed this view, suggesting a gender effect in this process. Girls who subsequently developed schizophrenia scored lower in this item compared with girls who did not, whereas boys who developed schizophrenia scored higher in self-esteem and school motivation than other boys who did not. Peer relationships and social comparison play a particularly strong role in shaping self-esteem for girls during adolescence, serving as important emotional buffers compared with boys.^29^ When these networks are disrupted, declines in self-esteem tend to be more pronounced for girls. This vulnerability coincides with a developmental period marked by increased sensitivity to social evaluation and gendered expectations, often leading to emotional withdrawal and reduced agency.^30^ Our results could be related to the proposal of a differential premorbid developmental trajectory towards psychosis in boys and girls, with boys exhibiting externalising symptoms and girls presenting with more internalising symptoms.^31^ These findings may therefore reflect an interaction between these normative processes and early psychotic vulnerability, reinforcing the need for gender-sensitive approaches to early risk detection. Our results also echo findings from the first-episode OPUS trial, which showed that women had worse self-esteem than men despite presenting better functioning,^32^ and a meta-analysis showing a small difference in self-esteem favouring males.^33^ One could hypothesise that the observed premorbid low self-esteem in girls might also be related to the self-reported lower quality of life that women with schizophrenia experience compared with men,^34^ which does not fit with the better response to treatment that women have.^35^ In the control group, gender differences in self-esteem and school motivation were minimal, contrasting with the marked divergence observed among children who later developed schizophrenia: girls showed markedly lower self-esteem and motivation, whereas boys scored slightly higher than their peers. This suggests that the gender-specific pattern is unique to the premorbid trajectories of schizophrenia and not attributable to general population trends, underscoring the importance of gender-sensitive approaches in early risk detection. School interventions that incorporate psychoeducational components focused on developing self-esteem and self-efficacy, or targeting internalising symptoms, could be key psychoeducational tools to prevent the worsening of these traits in at-risk girls.^36^ We should also consider that our study examined the premorbid period of patients, which might span many years before the onset of schizophrenia. As such, our findings in boys do not mean that low self-esteem and school motivation could be significant proximal antecedents to the development of a first episode of schizophrenia.

Contrary to our initial hypothesis, we found a higher growth mindset score in participants who later developed schizophrenia than in those who did not. To our knowledge, a growth mindset has not been assessed in schizophrenia or its premorbid states before. We replicated previously observed associations between growth mindset and gender (more fixed in girls)^37^ and academic performance (growth mindset associated with higher academic performance),^26^ which suggests that the instrument accurately measured this construct in the children.

Our findings could be interpreted within the framework of the Differential Susceptibility Model, which posits that individuals’ sensitivity to environmental influences, both negative and positive, varies.^38^ Growth mindset, i.e. the belief in one’s ability to develop abilities through effort, could represent a positive adaptive response to the greater genetic susceptibility of individuals who develop psychopathologies in adulthood.^39^ Rather than being opposing forces, these adaptive responses and sensitivities may represent interconnected dimensions of an individual’s environmental plasticity. Increased sensitivity to the environment may lead to pathological outcomes under adverse conditions, as well as the activation of beneficial internal resources in response to positive environments across different stages of life.^40^ Regardless of whether those who later develop schizophrenia can be considered children with heightened sensitivity to social contexts, these findings highlight the urgent need for further research to unravel the still unknown relationship between growth mindset and the underlying mechanisms of premorbid trajectories of severe psychiatric disorders. No direct measures of environmental sensitivity or genetic susceptibility were available in our data-set.

An alternative interpretation is that a higher growth mindset among future patients could reflect exposure to cognitive changes in relatives, fostering a belief in change that includes deterioration. This explanation is not directly testable with our data and should be interpreted as a hypothesis. In line with this framework, we observed that the positive association between academic performance and growth mindset typically seen in healthy youth was reversed among those who later developed schizophrenia: higher GPA was linked to weaker beliefs in the malleability of abilities. This pattern may reflect increased cognitive effort, self-doubt or early alterations in self-referential and motivational processing.^41^ Conversely, lower-achieving children showing a higher growth mindset may represent a compensatory form of adaptation, as growth mindset has been shown to buffer the negative emotional impact of academic stress.^42^ Together, these findings suggest that heightened environmental sensitivity in this group may manifest as both vulnerability and adaptive motivational plasticity, depending on contextual demands.

### Limitations and strengths

Our study is not without limitations. First, our sample included a high proportion of patients who were started on clozapine, signalling a more severe spectrum of the disorder. We did not find any significant differences in premorbid measures when comparing individuals who were and were not prescribed clozapine. However, caution is needed when extrapolating our findings to a less severe group of patients, because of the low statistical power in this subgroup.

Second, although our outcomes were measured in a premorbid stage of schizophrenia, we cannot ensure that these were unaffected by early symptoms of the disease. In particular, assessments conducted in mid-adolescence may overlap with subtle or subclinical prodromal phenomena, which could influence self-reported socioemotional measures. Additionally, some of our controls might have developed psychosis and not be identified either by the clinical services or required clozapine, or might have died, biasing our case–control comparisons. However, the magnitude of both events in young people is expected to be low, unlikely changing substantially our results. Nevertheless, control misclassification would generally bias estimates toward the null, meaning true premorbid differences could be underestimated.

Third, the absence of data on participants’ familial psychiatric history may influence both genetic and environmental risk. For example, associations between parenting practices and later schizophrenia could be confounded by mental health conditions in caregivers. Future research should integrate detailed family clinical information to enable more precise modelling of risk and protective factors.^43^ The absence of family history also limits our ability to interpret whether observed differences reflect inherited liability, early environmental factors or interactions between the two.

Fourth, growth mindset and self-efficacy are related but distinct constructs, and some overlap is reflected in the items available in national assessments. Our selection was based on maximising longitudinal coverage alongside retaining items aligned with growth mindset theory. The two items in the 2012 surveys closely match Dweck’s original scale.^4,14^ The items included in later years match the growth mindset instrument implemented by the California Office to Reform Education districts surveys in California. Restricting analyses to only one type of instrument would have severely reduced the sample size.

Fifth, the diagnosis was made clinically, but not in a standardised manner. Although clinical diagnoses reflect real-world practice, variability across clinicians and settings may introduce noise into case ascertainment; however, such non-differential misclassification is likely to reduce, not inflate, observed associations. National tests are administered to all students, but some may not respond to certain questions or be absent on the assessment day.

Sixth, findings for growth mindset may have limited generalisability because of the restricted sample in which this construct was assessed. Because data availability was higher among students with a higher GPA, and GPA tends to correlate positively with growth mindset and self-esteem/motivation, observed case–control contrasts for these outcomes are likely conservative (biased toward the null); where an outcome correlates negatively with GPA, bias could go in the opposite direction.

Finally, our constructs are based on questions related to academic performance. Therefore, questions on parental support, self-esteem and motivation, and growth mindset were either explicitly related to school life or, even if framed in a more general manner, were asked in an educational context. Despite this, educational dimensionality complements the existing literature, which has analysed the general dimensions of these socioemotional outcomes. Emotional aspects and parental support within an educational context may also inform an important outcome in people with schizophrenia – educational attainment – which has remained persistently low despite medical advances.^17^

Our findings highlight the potential of educational settings as strategic platforms for cost-effective preventive interventions, particularly around parental support. Given the observational design of this study, we cannot infer that strengthening protective factors prevents the onset of psychosis. Nevertheless, our findings underscore the importance of school policies that promote emotional well-being, teacher training and family engagement; strategies that benefit all students and may provide additional support for those with higher psychosocial vulnerability.^44^

In summary, we showed that participants who later developed schizophrenia reported lower perceived educational parental support during childhood and exhibited a high growth mindset. Girls experienced lower self-esteem and school motivation, whereas boys did not. These results may inform additional research on early identification and preventive strategies in schizophrenia.

## Supporting information

Vasquez-Nuñez et al. supplementary material Vasquez-Nuñez et al. supplementary material

## Data Availability

The data supporting this study can be downloaded at the school level from the following link: School Data Download. To access the database containing individual test scores, student, parent and teacher questionnaires, please visit the transparency platform and complete the Public Information Request: Transparency Platform.

## Supplementary material

The supplementary material is available online at https://doi.org/10.1192/bjo.2026.11004
