# Supplementary material for: Childhood socioemotional development and growth mindset preceding schizophrenia: case–control study using prospectively collected data
Source: BJPsych Open. 2026 Apr 6;12(3):e99. doi: 10.1192/bjo.2026.11004 (PMC13122333; doi:10.1192/bjo.2026.11004)
Supplement: Vasquez-Nuñez et al. supplementary material [file S2056472426110047sup001.pdf]

**Supplementary Material**

# Childhood socioemotional development and growth mindset preceding schizophrenia: A case-control study using prospectively collected data

Javiera Vasquez-Nuñez, José Conejeros, Camila Díaz-Dellarossa, Cristian Mena, Juan Undurraga, Alfonso González, Rubén Nachar, Susana Claro, Eduardo A. Undurraga*, and Nicolás Crossley*

## Contents

*Figure S1. Data Anonymization Process* ........ *2*

*Figure S2. Participant Attrition Flowchart.* ........ *3*

*Figure S3. Interaction plot for Growth Mindset showing the effect of Diagnosis of Schizophrenia and Grade Point Average (GPA).* ........ *4*

*Internal consistency constructs by cohort 2012-2019 and level* ........ *5*

- Table S1. Internal consistency of the construct of Self-Esteem and School Motivation for each level-cohort ........ 5
- Table S1a. Questions used to build a Z-scored construct of Self-Esteem and School Motivation 6
- Table S2. Internal consistency of construct Perceived Educational Parental Support for each level-cohort ........ 8
- Table S2a. Questions used to build a Z-scored construct of Perceived Educational Parental Support ........ 8
- Table S3. Internal consistency of the Growth Mindset Support construct for each level-cohort .. 9
- Table S3a. Questions used to build a Z-scored Growth Mindset construct ........ 9

*Table S4. Eligible, included, and not included observations, size, and academic performance* ........ *10*

*Table S5. Mixed models for Perceived Educative Parental Support, Self-esteem and School Motivation, Growth Mindset - Cohort 2012-2019 4th, 8th, and 10th grades* ........ *11*

*STROBE Statement—checklist of items that should be included in reports of observational studies* ....... *12*

* Joint senior authorship.

Corresponding authors: Nicolás A. Crossley, Department of Psychiatry, School of Medicine, Pontificia Universidad Católica de Chile, Diagonal Paraguay 362, Santiago, Región Metropolitana 8330077, Chile; ncrossley@uc.cl; and Eduardo A. Undurraga, School of Government, Pontificia Universidad Católica de Chile, Av. Vicuña Mackenna 4860, Macul, Santiago, Región Metropolitana 7820436, Chile; eundurra@uc.cl.

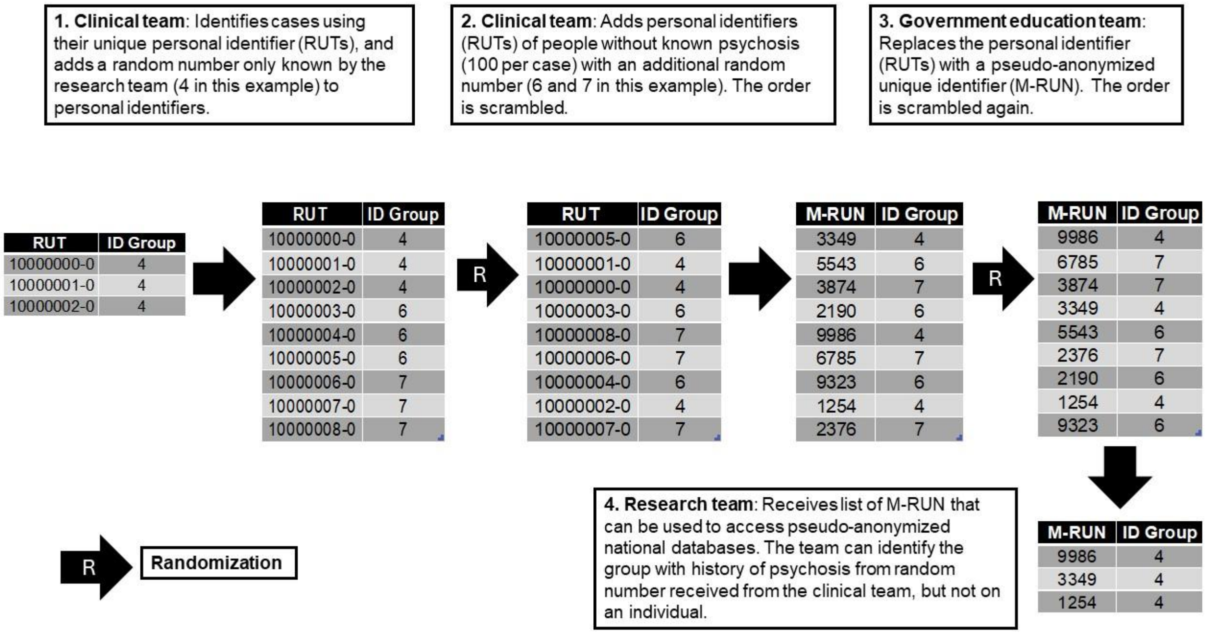


**Figure S1. Data Anonymization Process**

Note. RUT is the national ID number in Chile. M-RUN is the unique pseudo-anonymized identifier used in educational databases in Chile available for researchers. The process from RUT to M-RUN is only managed by the Ministry of Education.

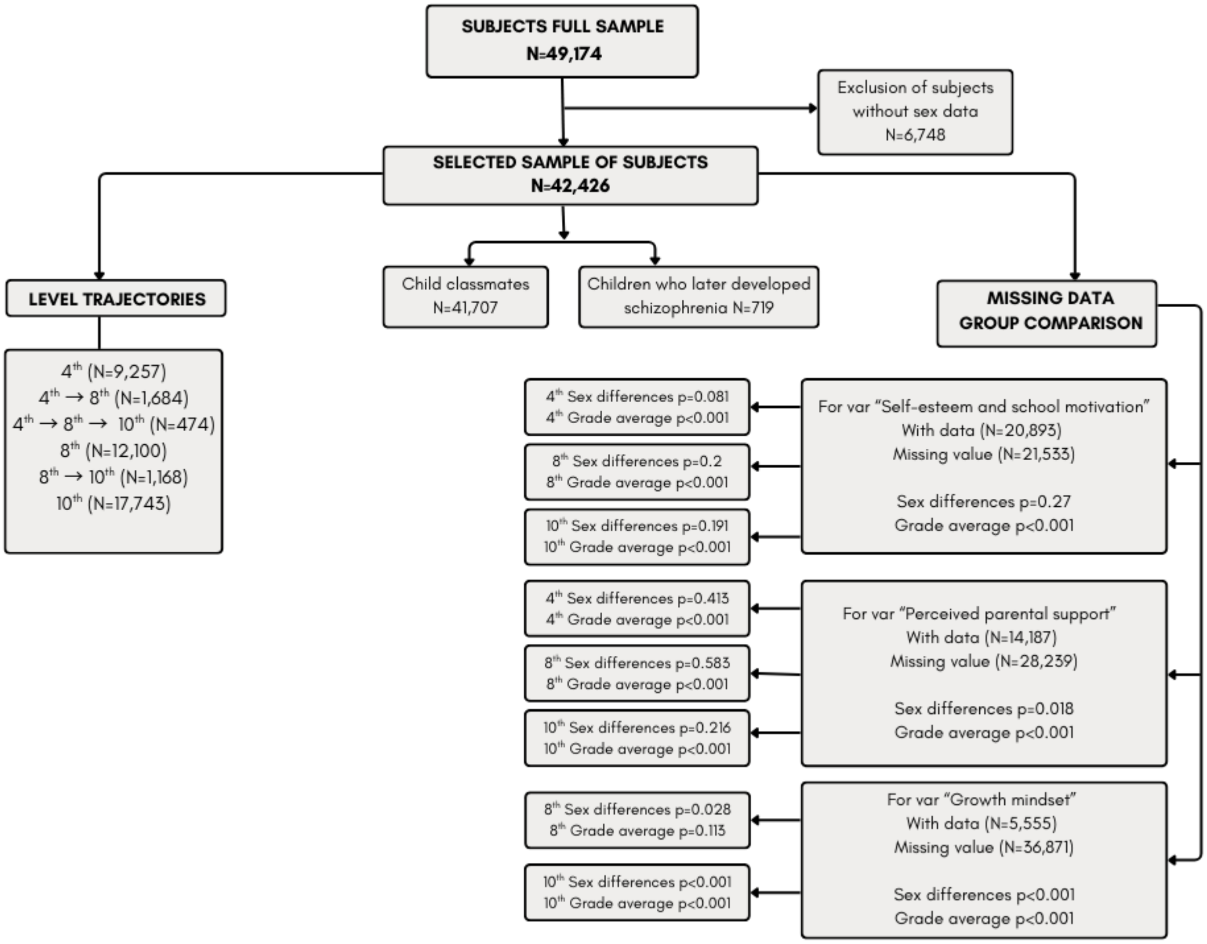


**Figure S2. Participant Attrition Flowchart.**

Note. T-tests were performed to estimate differences in grade average. Chi-square tests were performed to estimate differences in sex. To analyze differences in grade average between participants who had and did not have data for outcomes, an average grade average was estimated in cases where the participant was in more than one level.

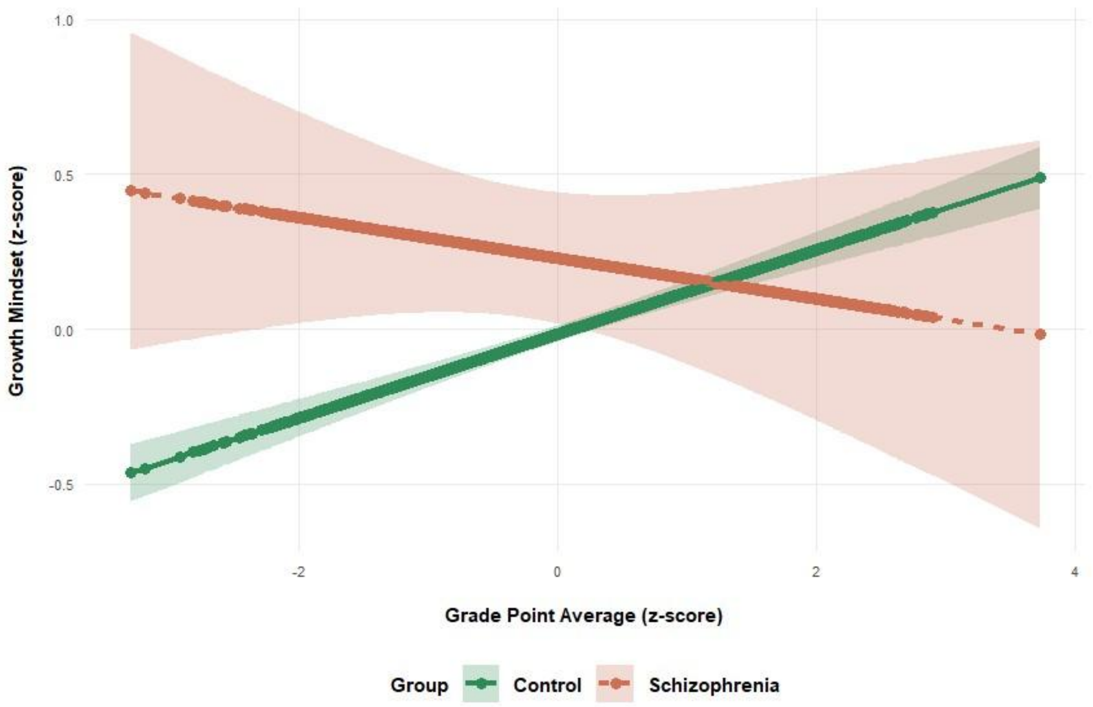


**Figure S3. Interaction plot for Growth Mindset showing the effect of Diagnosis of Schizophrenia and Grade Point Average (GPA).**

Note. The solid line and green confidence interval represent child controls. The solid line and light red confidence interval represent children who later developed schizophrenia. The colour areas (green for child controls and light red for children who later developed schizophrenia) represent the 95% confidence intervals.

## Internal consistency constructs by cohort 2012-2019 and level

**Table S1. Internal consistency of the construct of Self-Esteem and School Motivation for each level-cohort**

| Level | Cohort | Question | Cronbach's Alpha |
|---|---|---|---|
| 4th | 2012 | a1 a3 a6 a7 a10 a11 b13 b14 b15 b16 b17 b18 | 0.81 |
| 10th | 2012 | a1 a2 a3 a4 a6 a7 a12 a10 a13 a14 a39 b13 b14 b15 b17 b18 | 0.83 |
| 4th | 2013 | a1 a3 a5 a7 a8 a10 a11 b15 b16 b17 | 0.74 |
| 8th | 2013 | a1 a3 a4 a6 a10 a12 a13 a39 b14 b16 b15 b17 b19 | 0.81 |
| 10th | 2013 | a1 a2 a3 a4 a6 a10 a12 a13 a39 b14 b15 b16 b17 b19 | 0.81 |
| 4th | 2014 | a1 a2 a5 a7 a8 a10 a11 b15 b16 b17 | 0.76 |
| 8th | 2014 | a1 a2 a3 a4 a6 a7 a12 a13 a15 a39 b14 b15 b16 b17 b19 | 0.83 |
| 10th | 2014 | a1 a2 a3 a4 a6 a7 a12 a13 a15 a39 b14 b15 b16 b17 b19 | 0.83 |
| 4th | 2015 | a2 a5 a7 a10 a11 a16 a17 | 0.62 |
| 8th | 2015 | a3 a6 a7 a10 a12 a13 a15 a17 a18 a39 | 0.81 |
| 10th | 2015 | a3 a6 a7 a10 a12 a13 a15 a17 a18 a39 | 0.81 |
| 4th | 2016 | a2 a3 a10 a17 a19 a22 a23 a24 a25 | 0.73 |
| 10th | 2016 | a3 a7 a10 a39 a13 a15 a17 a18 a28 a29 | 0.81 |
| 4th | 2017 | a15 a17 a19 a20 a21 a22 a30 a31 | 0.80 |
| 8th | 2017 | a3 a7 a15 a17 a18 a19 a29 a30 a31 | 0.82 |
| 10th | 2017 | a3 a7 a15 a17 a18 a19 a29 a30 a31 a39 | 0.84 |
| 4th | 2018 | a15 a17 a19 a20 a21 a22 a30 a31 a34 a35 | 0.89 |
| 10th | 2018 | a3 a7 a15 a17 a18 a19 a29 a30 a31 a39 | 0.85 |
| 8th | 2019 | a3 a20 a15 a17 a19 a20 a28 a37 a38 | 0.79 |

Note. The questions indicated are those that the Ministry of Education considers for each of our constructs (outcomes).

**Table S1a. Questions used to build a Z-scored construct of Self-Esteem and School Motivation**

| Question Code | Original in Spanish | English Translation |
|---|---|---|
| **a1** | Estoy contento con las notas que logro sacarme. | I am happy with the grades I get. |
| **a2** | Cuando me va mal en un ramo o asignatura, me rindo fácilmente. | When I do poorly in a subject, I give up easily. |
| **a3** | Me entretiene aprender cosas nuevas. | I enjoy learning new things. |
| **a4** | Me preocupo de preguntarle al profesor cuando explica algo en clase y no lo entiendo. | I make sure to ask the teacher when they explain something in class, and I do not understand it. |
| **a5** | Siento que soy más capaz de aprender que el resto de mis compañeros de curso. | I feel I am more capable of learning than my classmates. |
| **a6** | Aunque sea difícil una materia, con estudio creo que puedo entenderla. | Even if a subject is hard, I think I can understand it with studying. |
| **a7** | Siempre hago mis tareas. | I always do my homework. |
| **a8** | Me cuesta concentrarme y poner atención en clases. | I have trouble concentrating and paying attention in class. |
| **a10** | Hago las tareas aunque sean difíciles. | I do my homework even if it is difficult. |
| **a11** | Todo lo que hago me resulta mal. | Everything I do turns out badly. |
| **a12** | Si falto a clases, me preocupo de conseguir la materia. | If I miss a class, I make sure to get the material I missed. |
| **a13** | Si me saco una mala nota, estudio más para la próxima prueba. | If I get a bad grade, I study more for the next test. |
| **a14** | Me cuesta entender lo que me enseñan en clases. | I find it hard to understand what they teach in class. |
| **a15** | Me gusta estudiar. | I like studying. |
| **a17** | Sé que me puedo sacar buenas notas si me esfuerzo. | I know I can get good grades if I try hard. |
| **a18** | Siempre trato de entender bien lo que me enseñan en clases. | I always try to understand well what they teach in class. |
| **a19** | Sé que siempre puedo hacer bien mis tareas. | I know I can always do my homework well. |
| **a20** | Puedo aprender con facilidad y rapidez. | I can learn easily and quickly. |
| **a21** | Sé que soy un buen estudiante. | I know I am a good student. |
| **a22** | Sé que puedo terminar el año con un buen promedio de notas. | I know I can finish the year with good grades. |
| **a23** | Hago las tareas porque quiero ser un buen estudiante. | I do my homework because I want to be a good student. |
| **a24** | Cuando me aburro no termino las tareas. | When I get bored, I don’t finish my homework. |
| **a25** | Me gusta hacer trabajos en grupo con mis compañeros. | I like doing group projects with my classmates. |

| | | |
|---|---|---|
| **a28** | Pongo atención en todas las clases, aunque algunas no me gusten. | I pay attention in all classes, even if I do not like some of them. |
| **a29** | Aprender lo que me enseñan en la escuela es muy importante para mí. | Learning what they teach at school is very important to me. |
| **a30** | Me esfuerzo por ser un buen estudiante. | I try hard to be a good student. |
| **a31** | Me esfuerzo para que me vaya bien en todas las asignaturas. | I try hard to do well in all subjects. |
| **a34** | Estoy seguro que cada día puedo aprender más. | I am sure I can learn more every day. |
| **a35** | Estoy seguro que puedo lograr mis metas. | I am sure I can achieve my goals. |
| **a37** | Me agrada hacer las actividades que nos dan en clase. | I enjoy doing the activities we are given in class. |
| **a38** | Cuando algo me interesa, busco más información para saber más de lo que me enseñaron en clases. | When something interests me, I look for more information to learn more about what was taught in class. |
| **a39** | La mayoría de las cosas que hago en mi establecimiento me resultan bien. | Most of the things I do at school turn out well. |
| **b13** | Hago bien mis tareas. | I do my homework well. |
| **b14** | Cuando sea grande voy a lograr todo lo que me proponga. | When I grow up, I will achieve everything I set my mind to. |
| **b15** | Soy inteligente. | I am intelligent. |
| **b16** | Puedo hablar bien delante de mi curso. | I can speak well in front of my class. |
| **b17** | Normalmente recuerdo lo que aprendo. | I usually remember what I learn. |
| **b18** | Los profesores piensan que soy capaz de aprender. | Teachers think I am capable of learning. |
| **b19** | Mis compañeros piensan que tengo buenas ideas. | My classmates think I have good ideas. |

**Table S2. Internal consistency of construct Perceived Educational Parental Support for each level-cohort**

| Level | Cohort | Question | Cronbach's Alpha |
|---|---|---|---|
| 4th | 2012 | b1 b2 b4 b6 b7 | 0.67 |
| 10th | 2012 | b7 b10 | 0.66 |
| 4th | 2013 | b1 b2 b4 b6 b7 | 0.66 |
| 8th | 2013 | b7 b10 | 0.63 |
| 10th | 2013 | b7 b10 | 0.66 |
| 4th | 2014 | b1 b2 b4 | 0.70 |
| 8th | 2014 | b7 b10 | 0.67 |
| 10th | 2014 | b7 b10 | 0.64 |
| 4th | 2016 | b4 b7 b11 b29 b30 | 0.76 |
| 10th | 2016 | b6 b7 b10 b11 | 0.90 |
| 4th | 2017 | b29 b30 | 0.85 |

Note. The questions indicated are those that the Ministry of Education considers for each of our constructs (outcomes).

**Table S2a. Questions used to build a Z-scored construct of Perceived Educational Parental Support**

| Question Code | Original in Spanish | English Translation |
|---|---|---|
| **b1** | Me explica la materia que no entiendo. | They explain the material I do not understand. |
| **b2** | Me ayuda a estudiar. | They help me study. |
| **b4** | Me ayuda a hacer las tareas o trabajos (pero sin hacérmela completamente). | They help me with my homework or projects (but without doing it completely for me). |
| **b6** | Sabe o se entera de las notas que tengo. | They know or find out about the grades I get. |
| **b7** | Me felicita cuando me saco buenas notas. | They congratulate me when I get good grades. |
| **b10** | Está dispuesto a ayudarme cuando tengo problemas con una materia o necesito resolver una tarea. | They are willing to help me when I have problems with a subject or need to complete a task. |
| **b11** | Me felicita cuando me esfuerzo en la escuela | They congratulate me when I put effort into school. |
| **b29** | Mis padres me dicen que soy un buen estudiante. | My parents tell me that I am a good student. |
| **b30** | Mis padres me dicen que soy capaz de aprender. | My parents tell me that I am capable of learning. |

**Table S3. Internal consistency of the Growth Mindset Support construct for each level-cohort**

| Level | Cohort | Question | Cronbach Alpha |
|---|---|---|---|
| 10th | 2012 | b20 b21 | 0.85 |
| 8th | 2017 | b20 b22 b23 | 0.62 |
| 10th | 2017 | b20 b22 b23 | 0.66 |
| 10th | 2018 | b20 b22 b23 | 0.73 |
| 8th | 2019 | b20 b22 b23 | 0.62 |

Note. The questions indicated are those that the Ministry of Education considers for each of our constructs (outcomes).

**Table S3a. Questions used to build a Z-scored Growth Mindset construct**

| Question Code | Original in Spanish | English Translation |
|---|---|---|
| **b20** | La inteligencia es algo que no se puede cambiar mucho. | Intelligence is something that cannot be changed much. |
| **b21** | Se pueden aprender cosas nuevas, pero no se puede cambiar la inteligencia de una persona. | You can learn new things, but you cannot change a person's intelligence. |
| **b22** | Ponerme desafíos no me hará más inteligente. | Challenging myself will not make me smarter. |
| **b23** | Hay ciertas cosas que simplemente no soy capaz de aprender. | There are certain things I am simply not capable of learning. |

**Table S4. Eligible, included, and not included observations, size, and academic performance**

| Variable | Elegible N (% Female) | Included N (%Female) | No included N (%Female) | Mean GPA Included | Mean GPA No included | P-value |
|---|---|---|---|---|---|---|
| **All** | | | | | | |
| All | 27,678 (47.34) | 22,074 (47.60) | 5,604 (46.32) | .063 | -.283 | .000 |
| Schizophrenia | 695 (32.66) | 438 (34.24) | 257 (29.96) | -.194 | -.706 | .000 |
| Control | 26,983 (47.72) | 21,636 (47.87) | 5,347 (47.11) | .068 | -.262 | .000 |
| **Perceived Educational Parental Support** | | | | | | |
| All | 18,736 (46.58) | 14,540 (46.77) | 4,196 (45.94) | .068 | -.258 | .000 |
| Schizophrenia | 483 (32.09) | 304 (34.53) | 179 (27.93) | -.175 | -.653 | .000 |
| Control | 18,253 (47.95) | 14,236 (47.03) | 4,017 (46.75) | .073 | -.240 | .000 |
| **Self-esteem and School Motivation** | | | | | | |
| All | 27,678 (47.34) | 22,057 (47.60) | 5,621 (46.30) | .063 | -.282 | .000 |
| Schizophrenia | 483 (46.99) | 304 (49.34) | 179 (43.01) | -.194 | -.706 | .000 |
| Control | 26,983 (47.72) | 21,619 (47.87) | 5,364 (47.09) | .068 | -.261 | .000 |
| **Growth Mindset** | | | | | | |
| All | 7,206 (49.75) | 5,615 (50.11) | 1,591 (48.46) | .072 | -.302 | .000 |
| Schizophrenia | 157 (31.84) | 88 (28.40) | 69 (36.23) | -.227 | -.801 | .010 |
| Control | 7,049 (50.14) | 5,527 (50.46) | 1,522 (49.01) | .077 | -.277 | .000 |

Note. The number of eligible observations corresponds to participants who were eligible for the standardised testing. The number of included observations corresponds to participants with data for at least one indicator used to construct each variable. The number of observations not included refers to participants with no available data on any relevant indicator.

**Table S5. Mixed models for Perceived Educative Parental Support, Self-esteem and School Motivation, Growth Mindset - Cohort 2012-2019 4th, 8th, and 10th grades**

| Variable | A. Perceived Educational Parental Support | | B. Self-esteem and School Motivation | | C. Growth Mindset | |
|---|---|---|---|---|---|---|
| | Model 1SA | Model 2SA | Model 1SB | Model 2SB | Model 1SC | Model 2SC |
| Schizophrenia | -0.215** | -0.322* | 0.153** | 0.0355 | 0.342** | 0.418 |
| | (0.0711) | (0.132) | (0.0554) | (0.106) | (0.128) | (0.270) |
| Female | -0.0210 | -0.0242 | 0.0251* | 0.0196 | -0.0638* | -0.0659* |
| | (0.0166) | (0.0165) | (0.0126) | (0.0125) | (0.0265) | (0.0263) |
| Ed. Level 4th | 0.0043 | 0.0035 | 0.0019 | 0.000989 | | |
| | (0.0231) | (0.0231) | (0.0193) | (0.0193) | | |
| Ed. Level 8th | 0.0039 | 0.0035 | 0.0059 | 0.00542 | 0.0006 | 0.0008 |
| | (0.0213) | (0.0213) | (0.0148) | (0.0148) | (0.0462) | (0.0462) |
| Schizophrenia x Female | -0.175 | | -0.315** | | -0.181 | |
| | (0.120) | | (0.0939) | | (0.234) | |
| Year GPA | 0.076** | 0.0764** | 0.403** | 0.403** | 0.133** | 0.132** |
| | (0.0086) | (0.00861) | (0.0064) | (0.0064) | (0.0134) | (0.0134) |
| Clozapine Prescription | | 0.0572 | | 0.0105 | | -0.154 |
| | | (0.146) | | (0.117) | | (0.293) |
| Constant | 0.0102 | 0.0121 | -0.0379* | -0.0348* | 0.0143 | 0.0156 |
| | (0.0198) | (0.0197) | (0.0174) | (0.0174) | (0.0239) | (0.0239) |
| Log-Likelihood | -20310.24 | -20311.22 | -28981.12 | -28986.73 | -7745.16 | -7745.322 |
| AIC | 40650.49 | 40652.44 | 57998.24 | 58009.47 | 15516.32 | 15516.64 |
| Observations | 14,540 | 14,540 | 22,057 | 22,057 | 5,615 | 5,615 |
| N Groups Schools | 417 | 417 | 550 | 550 | 165 | 165 |
| N Groups Students | 14,242 | 14,242 | 21,050 | 21,050 | 5,556 | 5,556 |
| Var: School (Random Intercept) | 2.87e-07 | 6.33e-11 | 2.71e-11 | 1.24e-09 | 3.37e-11 | 2.95e-07 |
| Var: Student (Random Intercept) | 0.5283 | 0.5289 | 0.5807 | 0.5816 | 0.9470 | 0.9471 |
| Var: Residual | 0.8242 | 0.8239 | 0.6930 | 0.6925 | 0.2223 | 0.2222 |

Notes. ** *P<0.001* * *P<0.05*.

N = 14,540 for Perceived Educative Parental Support models; 22,057 for Self-Esteem and School Motivation models; 5,615 for Growth Mindset models.

Robust standard errors are in parentheses.

Ed. = Educational; Year GPA = Grade Point Average; AIC = Akaike Information Criterion.

**STROBE Statement—checklist of items that should be included in reports of observational studies**

| | Item No. | Recommendation | Page No. |
|---|---|---|---|
| **Title and abstract** | 1 | (*a*) Indicate the study's design with a commonly used term in the title or the abstract | Page 1 |
| | | (*b*) Provide in the abstract an informative and balanced summary of what was done and what was found | Page 2 |
| **Introduction** | | | |
| Background/rationale | 2 | Explain the scientific background and rationale for the investigation being reported | Page 3 |
| Objectives | 3 | State specific objectives, including any prespecified hypotheses | Page 4 |
| **Methods** | | | |
| Study design | 4 | Present key elements of study design early in the paper | Page 5 |
| Setting | 5 | Describe the setting, locations, and relevant dates, including periods of recruitment, exposure, follow-up, and data collection | Page 5 to 6 |
| Participants | 6 | (*a*) *Cohort study*—Give the eligibility criteria, and the sources and methods of selection of participants. Describe methods of follow-up<br>*Case-control study*—Give the eligibility criteria, and the sources and methods of case ascertainment and control selection. Give the rationale for the choice of cases and controls<br>*Cross-sectional study*—Give the eligibility criteria, and the sources and methods of selection of participants | Page 5 |
| | | (*b*) *Cohort study*—For matched studies, give matching criteria and number of exposed and unexposed<br>*Case-control study*—For matched studies, give matching criteria and the number of controls per case | |
| Variables | 7 | Clearly define all outcomes, exposures, predictors, potential confounders, and effect modifiers. Give diagnostic criteria, if applicable | Page 6 to 7 |
| Data sources/ measurement | 8* | For each variable of interest, give sources of data and details of methods of assessment (measurement). Describe comparability of assessment methods if there is more than one group | Page 6 to 8 |
| Bias | 9 | Describe any efforts to address potential sources of bias | Page 7 to 9 and Figure S2 in SM** |
| Study size | 10 | Explain how the study size was arrived at | Page 5 to 6 |
| Quantitative variables | 11 | Explain how quantitative variables were handled in the analyses. If applicable, describe which groupings were chosen and why | Page 6 to 8 |
| Statistical methods | 12 | (*a*) Describe all statistical methods, including those used to control for confounding | Page 8 to 9 |
| | | (*b*) Describe any methods used to examine subgroups and interactions | Page 9 |
| | | (*c*) Explain how missing data were addressed | Page 5 to 7 and Table S4 in SM** |
| | | (*d*) *Cohort study*—If applicable, explain how loss to follow-up was addressed | |
| | | *Case-control study*—If applicable, explain how matching of cases and controls was addressed | Page 6 |
| | | *Cross-sectional study*—If applicable, describe analytical methods taking account of sampling strategy | |
| | | (*e*) Describe any sensitivity analyses | Table |

| | | | |
|---|---|---|---|
| | | | S5 in SM** |
| **Results** | | | |
| Participants | 13* | (a) Report numbers of individuals at each stage of study—eg numbers potentially eligible, examined for eligibility, confirmed eligible, included in the study, completing follow-up, and analysed | Table S4 in SM** |
| | | (b) Give reasons for non-participation at each stage | Page 5 to 6; Figure S2 in SM** |
| | | (c) Consider use of a flow diagram | Figure S2 in SM** |
| Descriptive data | 14* | (a) Give characteristics of study participants (eg demographic, clinical, social) and information on exposures and potential confounders | Page 10; Table 1; Table S4 in SM** |
| | | (b) Indicate number of participants with missing data for each variable of interest | Table S4; Figure S2 in SM** |
| | | (c) *Cohort study*—Summarise follow-up time (eg, average and total amount) | |
| Outcome data | 15* | *Cohort study*—Report numbers of outcome events or summary measures over time | |
| | | *Case-control study*—Report numbers in each exposure category, or summary measures of exposure | Table 1; Figure 1 |
| | | *Cross-sectional study*—Report numbers of outcome events or summary measures | |
| Main results | 16 | (*a*) Give unadjusted estimates and, if applicable, confounder-adjusted estimates and their precision (eg, 95% confidence interval). Make clear which confounders were adjusted for and why they were included | Page 10 to 11; Table 2 |
| | | (*b*) Report category boundaries when continuous variables were categorized | |
| | | (*c*) If relevant, consider translating estimates of relative risk into absolute risk for a meaningful time period | |
| Other analyses | 17 | Report other analyses done—eg analyses of subgroups and interactions, and sensitivity analyses | Table S5, Figure S2 in SM** |
| **Discussion** | | | |
| Key results | 18 | Summarise key results with reference to study objectives | Page 11 |
| Limitations | 19 | Discuss limitations of the study, taking into account sources of potential bias or imprecision. Discuss both direction and magnitude of any potential bias | Page 15 to 16 |
| Interpretation | 20 | Give a cautious overall interpretation of results considering objectives, limitations, multiplicity of analyses, results from similar studies, and other relevant evidence | Page 15 to 16 |
| Generalisability | 21 | Discuss the generalisability (external validity) of the study results | Page 15 to 17 |
| **Other information** | | | |
| Funding | 22 | Give the source of funding and the role of the funders for the present study and, if applicable, for the original study on which the present article is based | Page 17 |

Note. *Give information separately for cases and controls in case-control studies and, if applicable, for exposed and unexposed groups in cohort and cross-sectional studies.
**SM = Supplementary Material
